# Identifying factors likely to influence compliance with diagnostic imaging guideline recommendations for spine disorders among chiropractors in North America: a focus group study using the Theoretical Domains Framework

**DOI:** 10.1186/1748-5908-7-82

**Published:** 2012-08-31

**Authors:** André E Bussières, Andrea M Patey, Jill J Francis, Anne E Sales, Jeremy M Grimshaw

**Affiliations:** 1 Population Health Program, Ottawa Hospital Research Institute, University of Ottawa, Ottawa, Canada; 2 Departement Chiropratique, Université du Québec à Trois-Rivières, Trois-Rivières, Canada; 3 Clinical Epidemiology Program, Ottawa Hospital Research Institute – General Campus, Ottawa, Canada; 4 Health Psychology Group and Health Services Research Unit, University of Aberdeen, Foresthill campus, Aberdeen, UK; 5 School of Nursing, University of Michigan, Ann Arbor, USA; 6 Center for Clinical Management Research, VA Ann Arbor Health Care System, Ann Arbor, USA; 7 Faculty of Medicine, University of Ottawa, Ottawa, Canada; 8 Canada PRIme Plus is a collaboration of international researchers, Canada, UK

**Keywords:** Theoretical domains framework, Focus groups, Content analysis, Social/professional role and identity, Social influence, Chiropractors, Radiography, X-ray guidelines, Back pain

## Abstract

**Background:**

The Theoretical Domains Framework (TDF) was developed to investigate determinants of specific clinical behaviors and inform the design of interventions to change professional behavior. This framework was used to explore the beliefs of chiropractors in an American Provider Network and two Canadian provinces about their adherence to evidence-based recommendations for spine radiography for uncomplicated back pain. The primary objective of the study was to identify chiropractors’ beliefs about managing uncomplicated back pain without x-rays and to explore barriers and facilitators to implementing evidence-based recommendations on lumbar spine x-rays. A secondary objective was to compare chiropractors in the United States and Canada on their beliefs regarding the use of spine x-rays.

**Methods:**

Six focus groups exploring beliefs about managing back pain without x-rays were conducted with a purposive sample. The interview guide was based upon the TDF. Focus groups were digitally recorded, transcribed verbatim, and analyzed by two independent assessors using thematic content analysis based on the TDF.

**Results:**

Five domains were identified as likely relevant. Key beliefs within these domains included the following: conflicting comments about the potential consequences of not ordering x-rays (risk of missing a pathology, avoiding adverse treatment effects, risks of litigation, determining the treatment plan, and using x-ray-driven techniques contrasted with perceived benefits of minimizing patient radiation exposure and reducing costs; *beliefs about consequences*); beliefs regarding professional autonomy, professional credibility, lack of standardization, and agreement with guidelines widely varied ( *social/professional role & identity*); the influence of formal training, colleagues, and patients also appeared to be important factors ( *social influences*); conflicting comments regarding levels of confidence and comfort in managing patients without x-rays ( *belief about capabilities*); and guideline awareness and agreements ( *knowledge*).

**Conclusions:**

Chiropractors’ use of diagnostic imaging appears to be influenced by a number of factors. Five key domains may be important considering the presence of conflicting beliefs, evidence of strong beliefs likely to impact the behavior of interest, and high frequency of beliefs. The results will inform the development of a theory-based survey to help identify potential targets for behavioral-change strategies.

## Background

Diagnostic imaging is commonly used in the management of musculoskeletal problems to improve precision in diagnosis prior to treatment. However, overuse and misuse of imaging services for spine disorders has been reported in the medical [1-4] and chiropractic literature [5-10]. Current evidence suggests that routine radiography is unnecessary during the initial evaluation of uncomplicated back pain unless specific clinical indicators (red flags) are present [4]. A more conservative approach to the diagnostic evaluation is therefore advisable, both in terms of health risk and resource allocation [11,12].

Clinical practice guidelines (CPGs) aim to describe appropriate care based on the best-available scientific evidence and broad consensus, while promoting efficient use of resources [13]. Although evidence-based diagnostic imaging guidelines for spinal disorders are available [14-16], chiropractors are divided on whether these guidelines apply to them [5,17-20]. Wide variations in lumbar spine x-ray ordering have been reported in North America, ranging from 12% to 26% of patients presenting with low back pain [18,21-23] to well over 55% [5,6,24-27]. The Diagnostic Imaging Guidelines for Adult Spine Disorders (DIGASD) were recently developed to assist clinical decision making and encourage more selective use of imaging studies by chiropractors and other primary healthcare professionals [28].

While CPGs can encourage providers to practice evidence-based care [29,30], passive dissemination of guidelines is unlikely by itself to lead to optimal practice [31,32]. By themselves, CPGs cannot overcome the multitude of barriers to clinician adherence [33]. Several competing beliefs can prevent practitioners from using best evidence. Identifying determinants of specific clinical behavior likely to influence the implementation of practice guidelines is a recommended initial step to tailor interventions to improve patient care [34-37].

Studies exploring factors influencing use of lumbar x-rays for low-back pain patients have generally focused on physicians, and both clinical and nonclinical factors have been found to be associated with test ordering [38-46]. Fewer studies have examined factors that affect chiropractors’ decisions to order x-rays [6,19,20,39,47] (see Additional file  1). We are aware of no studies comparing chiropractors’ beliefs about the use of x-rays in the American and Canadian settings. Furthermore, very few studies have used a theory-based approach to identify barriers and facilitators to behavior change among chiropractors [48]. Theories can provide a framework for interpreting and predicting behavior, help tailor interventions to improve the likelihood of successful change, and can help evaluate potential causal mechanisms [49-54].

Psychological theories explaining behavior and behavior change are numerous and share overlapping constructs [55-57]. To maximize the accessibility and usefulness of psychological theories to researchers involved in evidence-based practice implementation, Michie and colleagues mapped 128 constructs from 33 theories and identified 12 discrete domains [58]. The Theoretical Domains Framework (TDF) covers a broad spectrum of individual and organizational theories, thereby limiting the risk of omitting important areas when exploring factors that may impact decision making regarding the use of evidence-based care in clinical practice. We used the TDF to explore chiropractors’ beliefs about management of low-back pain without imaging in two countries. This article is one in a series of articles documenting the development and use of the TDF to advance the science of implementation research. The series’ introductory article [59] provides an overview of the articles contained in the TDF Series.

## Methods

### Design

We conducted six focus groups based on the TDF with chiropractors in America and Canada. The interactive nature of focus groups can promote synergy among participants and allows exploration of collective memories, positions, ideology, practices, and desires among specific groups of people [60]. Focus groups are particularly useful when group norms and cultural values of particular groups are of interest and to explore the degree of consensus on a given topic [61,62].

### Context

Participants were identified from the American Specialty Health (ASH) Network service lists from two states (California and Georgia) that had differing x-ray–ordering rates and professional associations in Ontario and Quebec, Canada. The ASH Network provides complimentary healthcare, including chiropractic services, across the United States [27]. The ASH Network assigns chiropractors to one of six levels based on quality performance indicators, such as inappropriate or high x-ray–ordering practice. Each level defines the number of patient visits and services permitted before verification of medical necessity is required. Providers can move up or down in tier level following retrospective annual reviews based on guidelines compliance.

### Participants

Participants were licensed chiropractors in full-time practice and/or had prolonged experience as chiropractic educators. A purposive sample was drawn from the provider organizations to seek respondents across a spectrum (geographical area, chiropractic school attended, x-ray–ordering practice, years in practice, and expertise) to ensure that all viewpoints would be adequately represented.

## Materials

The specified target behavior was managing uncomplicated back pain without x-rays. An interview topic guide based on the TDF was developed [58] (see Additional file  2). Questions were informed by previously published work on the topic [43,54,63-67]. Probes were used where necessary for further clarification [68]. Face and content validity of the interview guide were initially assessed by experts in knowledge translation (JMG, JJF) and expert chiropractors, thereby ensuring that questions adequately covered each theoretical domain and were relevant to the clinicians. The number of questions ranged between two and seven for each of the 12 domains, for a total of 43 questions.

### Procedure

A total of 154 practitioners were invited to take part in focus groups consisting of four to six practitioners between February and July 2010. A customized letter invited care providers to participate. We followed up with the first 25 respondents via email or telephone. Those who agreed to participate were asked to review relevant recommendations of the DIGASD one to two weeks prior to the interview. The focus groups lasted between 60 and 90 minutes. They were digitally recorded, and field notes were taken by a nonparticipant observer during three of the six groups. It was not possible to arrange for a note taker to attend the other locations; however, the interviewer (AEB) made notes after each focus group. Data were transcribed verbatim and anonymized prior to analysis.

### Analysis

Transcripts were coded independently by two investigators (AEB and AMP) and disagreements formally resolved at each step. One author (JJF), a health psychologist, provided a critique of the analysis and interrogated the coding to ensure a robust and defensible coding of the data into beliefs and relevant domains.

We initially coded each utterance into relevant theoretical domains from the TDF onto an Excel spreadsheet. Utterances were counted twice if a participant gave a response similar to that of another participant. Coding was guided by our understanding of the constructs within a domain. Utterances unlikely to be relevant to lumbar x-ray–ordering practice were placed into a separate file for further analysis.

Then we linked utterance responses with specific beliefs. A specific belief is a statement that provides detail about the role of the domain in influencing the behavior [66]. These statements intended to convey a meaning that was common to multiple utterances. AEB generated specific beliefs from utterances that captured the core thought and continued this process for every utterance. Beliefs, coded as being similar or identical statements, were then grouped together according to their likelihood to either increase (*i.e.*, perceived barriers to guideline adherence), decrease ( *i.e.*, perceived to facilitate or help guideline adherence), or have no influence on x-ray–ordering behavior. Two to three emerging, overarching themes were proposed for each domain. Specific beliefs and overarching themes were reviewed for agreement by AMP and JJF.

Finally, we identified relevant domains based upon the following criteria: (1) presence of conflicting beliefs, (2) evidence of strong beliefs that were perceived to impact the behavior, and (3) high frequency of specific beliefs. All three criteria were weighed equally to judge relevance of the domains as they relate to influencing target behavior [66,69].

To assess whether or not we had achieved data saturation [70], we conducted concurrent data analysis and coding; themes were recurring after the third focus group, and no new themes emerged after the fifth focus group.

### Comparison of US and Canadian participants

American and Canadian participants were compared on (1) distribution of utterances within each domain, (2) identification of specific beliefs within domains, and (3) identification of relevant domains likely to influence lumbar x-ray–ordering behavior.

### Ethics

A signed collaborative agreement by the ASH Network was submitted to the Ottawa Hospital Research Ethics Board, Canada, who granted ethic approval.

## Results

### Characteristics of participants

Six focus group interviews were conducted (21 chiropractors), including two in California (n = 8), two in Georgia (n = 6), one in Ontario (n = 3), and one in Quebec (n = 4). The average age of participants was 44.2 years (SD ± 9.2), 28% (5/21) were females, and the average number of years in practice was 13.8 years (SD ± 7.9). Age, gender, and years in practice of our sample are representative of national averages in North America [71,72]. Sixteen participants were in full-time practice, five were academics or lecturers, and ASH provider tier level ranged from 3 to 6. Nearly 40% of interviewees reported ordering x-rays on over half of patients presenting with nonspecific back pain (*nature of the behaviors*; Table 1).

**Table 1:** Thematic content analysis based on the Theoretical Domains Framework (TDF)

**TDF domains**^**a**^	**Number of questions**	**Utterances**	**Reduce**^**b**^**(%)**	**Increase**^**c**^**(%)**	**No influence (%)**	**Specific beliefs**	**Themes**
*Nature of the behaviors*	2	88	45 (51.1)	35 (39.8)	8 (9.1)	7	Lumbar x-ray utilization
*Skills*	3	1	0 (0)	0 (0)	1 (100)	1	None identified
*Beliefs about capabilities*	3	106	70 (66)	31 (29.2)	5 (4.7)	5	Capabilities; ease of ordering test
*Motivation & goals*	5	50	28 (56)	17 (34)	5 (10)	4	Intrinsic motivations
*Beliefs about consequences*	4	334	93 (27.8)	211 (63.2)	30 (18.9)	15	Consequence of ordering lumbar x-rays; attitudes
*Environmental context and resources*	2	41	16 (39)	25 (61)	0	3	Environmental resource; Signs within the clinic
*Social influences*	2	128	37 (29)	62 (48)	19 (13.3)	6	Influence of others’ opinions (colleagues, patients); influence of organization and new literature
*Emotion*	2	4	1 (25)	1 (25)	2 (50)	2	Own emotions
*Knowledge*	7	86	39 (45.3)	33 (38.4)	14 (16.3)	6	Awareness of the guidelines; knowledge of evidence
*Memory, attention, and decision process*	4	77	67 (87)	10 (13)	0 (0)	8	Factors that influence decision; ease of decision
*Social/professional role &identity*	4	133	39 (29)	67 (50.4)	27 (20.3)	12	Professional role; professional norms; professional agreement
*Behavioral regulation*	5	91	91 (100)	0 (0)	0 (0)	12	Ways to reduce testing; barriers; organizational changes; post intentional behavior
Total	43	1,139	526 (46.2)	492 (43.2)	109 (9.6)	88	23

^a^TDF domains are presented in the order in which questions were asked in the focus groups. ^b^Utterances perceived to reduce x-ray utilization (facilitators). ^c^Utterances perceived to increase x-ray utilization (barriers).

### Key themes identified within relevant domains

Coding by two independent assessors identified 1,183 utterances representing 88 specific beliefs and 23 themes. Interrater reliability was not assessed as 100% consensus was achieved at each step. Key themes emerging from the focus groups with chiropractors were categorized within five theoretical domains: *beliefs about consequences*, *social/professional role and identity*, *social influences*, *beliefs about capabilities*, and *knowledge* (Table 1)*.*

Twenty-nine percent of utterances focused on the domain of *beliefs about consequences*, two-thirds of which related to factors increasing the likelihood of ordering x-rays. Distribution of utterances perceived to increase spine x-ray ordering (barriers) among participants was also greater for the domains of *social/professional role and identity* and *social influences* and marginally higher for *environmental context and resources*. Conversely, distribution of utterances likely to decrease x-ray ordering (facilitators) was higher for the domains of *beliefs about capabilities*, *knowledge*, and, to a lesser extent, *memory, attention, and decision process* and *behavioral regulation.* Domains of *skills* and *emotion* were very rarely mentioned.

We report the findings of key domains together with illustrative quotes grouped as “beliefs likely to increase x-ray ordering” and “beliefs likely to decrease x-ray ordering.” Each utterance is identified alphabetically to represent the location of focus groups (G: Georgia, C: California, O: Ontario, Q: Quebec) and numerically to represent specific focus groups. (Please see Additional file  3 for detailed coding of specific beliefs within all TDF domains.)

#### Beliefs about consequences

Fifteen specific beliefs mapped to this domain. Most participants indicated that the risk of missing a spinal pathology or anomaly were significant disadvantages of managing uncomplicated back pain without x-rays. Many participants took x-rays because of perceived risks of adverse treatment effects or fear of litigation, to help monitor patient conditions, and to improve patient compliance.

### Beliefs likely to increase x-ray ordering (barriers)

"“The problem is that we perform a service that could injure someone and we certainly want to know what we are dealing with before we start.” (G1)"

"“What about exposure to liability? If you don’t have an x-ray where you missed a diagnosis.” (C1)"

"“I think x-rays also help with the type of treatment I am going to use if there is an anomaly like a transitional segment or presence of a disease will change the way I treat the patient.” (O)"

Other participants commented on the financial motivation of routine x-ray, onsite imaging, and x-ray–driven techniques.

"“I think there might be a financial incentive to order x-rays, financial is definitely part of that. I might add as a whole you are pretending that you’re doing a more thorough job if you have onsite imaging.” (Q)"

In contrast, participants expressed a number of beliefs about the benefits of managing nonspecific back pain without x-rays, including minimizing ionizing radiation exposure to patients, reducing costs, minimizing adverse events from further investigation, and avoiding labelling of patients.

### Beliefs likely to decrease x-ray ordering (facilitators)

"“Benefits to not using x-rays are cost savings and minimizing patient radiation exposure.” (C1)"

"“… like any tests you may have equivocal findings and need further investigation that could lead to further medical procedures such as a biopsy and those carry their own risks, so there’s always that risk of complications related to further investigations.” (Q)"

"“[Other benefits] include avoiding creating anxiety to patients from incidental findings on routine x-rays…” (G2)"

Many providers believed guidelines were designed to further restrict practice. Furthermore, US participants suggested that provider networks restrict their autonomy if they don’t conform to their standards by assigning providers to lower tier levels. Maintaining the highest tier level to reduce administrative burden was perceived to be important by most participants:

"“…if you’re not top tier, you are so mired in paperwork and the reimbursement is so low.” (G2)"

"“Our management protocols tend to be dictated by [the third-party payers] reimbursement policy to a certain degree.” (G2)"

"“Your incentive is to keep the network … happy so you don’t get kicked off the panel.” (C2)"

"[Regarding third-party payers’ adoption of guidelines] “I don’t think that reducing ionizing radiation exposure is the argument, I think it comes down to cost reduction.” (G1)"

#### Social/professional role and identity

Twelve beliefs were grouped under the domain of *social/professional role and identity.* While many participants addressed the perceived need for professional autonomy, several others suggested that the lack of standardization, beliefs about the possibility of visualizing chiropractic subluxations on x-rays, and insufficient knowledge and skills of colleagues in the profession were problematic. In addition, many providers were concerned with the credibility of the chiropractic profession ( *e.g.*, importance of appropriate x-ray ordering). About half of all participants were trained to take x-rays routinely; however, only a minority believed that x-ray–driven techniques to establish treatment protocols were an integral part of current chiropractic practice. Agreement with available diagnostic-imaging guidelines widely varied among participants.

### Beliefs likely to increase x-ray ordering (barriers)

"“I want to be able to make my own decision; that’s why I got into chiropractic.” (G2)"

"“As a profession we take a lot of criticism… Some would say you’re not real doctors, you don’t take x-rays.” (G2)"

"“Some providers look for subluxations and the only way to tell for sure is to take an x-ray…” (G1)"

"“It’s just a guideline and you know it could help you in the decisional process but I don’t think it should be viewed as some holy bible of how to take x-rays, you know, it should be taken with a grain of salt.” (Q)"

### Beliefs likely to decrease x-ray ordering (facilitators)

"“If we reduced x-ray utilization rate, we could present ourselves to other professions and to the world as being efficient, doing the right thing for the right reason.” (G2)"

"“I don't do x-ray listing or x-rays based on a certain technique, I don’t think that’s necessary.” (C2)"

"“Those x-ray guidelines make a tremendous amount of sense to me.” (C1)"

#### Social influences

*Social influences*, including the influence of formal training, colleagues, patients, and the health management organization’s/institution’s guidelines, protocols, or requirements, appear to be important factors to consider.

### Beliefs likely to increase x-ray ordering (barriers)

"“…I remember the teacher saying ‘always x-ray the point of pain. So if a person comes in with first episode low-back pain or says my wrist has been really sore for three months, you better x-ray their wrist. I saw that as a ‘cover my butt’ or liability management.” (O)"

"“Patients who are worried of having something serious and are asking for x-rays do influence my decision to order films.” (Q)"

### Beliefs likely to decrease x-ray ordering (facilitators)

"“When the HMO [health management organization] started publishing their guidelines, I would say it did influence me to take less x-rays.” (C1)"

#### Beliefs about capabilities

Four specific *beliefs about capabilities* were identified, including participants’ self-efficacy, self-confidence, past experience, and clinical uncertainty in managing uncomplicated back pain without x-rays.

### Beliefs likely to increase x-ray ordering (barriers)

"“A disadvantage of not taking those films is not having the comfort level you need to treat patients.” (G2)"

"“I’ll use whatever tool I need including x-rays to get that information and make the right diagnosis.” (G1)"

### Beliefs likely to decrease x-ray ordering (facilitators)

“If during the history taking and physical examination the patient doesn’t show any red flags, I feel very confident to treat my patients without x-rays.” (Q)

#### Knowledge

The domain of *knowledge* was reflected by beliefs relating to the following: awareness of the existence of the x-ray guidelines, familiarity and agreement with the content of the guidelines, knowledge of conflicting guideline recommendations, and whether or not it was felt that the level of evidence was sufficient for proposed recommendations. Three participants admitted not being aware of the existence of the evidence-based guidelines, and three others had minimal knowledge of their content. In addition, some felt their colleagues were not up to date or had insufficient training in critical reading of the literature.

### Beliefs likely to increase x-ray ordering (barriers)

"“One of the fundamental problems that we have is that there’s not the depth of research in some of these areas for the guidelines to be terribly credible.” (O)"

"“How can two different organizations looking at the same data to generate guidelines could come up very with different recommendations? To me, this raises a lot of questions on the ethics of some people developing guidelines.” (C1)"

### Beliefs likely to decrease x-ray ordering (facilitators)

"“…most of us are willing to go along with what the science says and if the science says unequivocally that taking x-rays is to the patient’s detriment unless six visits have gone by and the whole situation…” (O)"

### Differences between American and Canadian participants

A secondary objective was to compare responses from American and Canadian participants. Specific beliefs and key domains were generally similar for both countries (Table 2). Nevertheless, important differences included perceived threat of litigation among US participants and ASH Network’s incentives to conform to evidence-based practice (*beliefs about consequences)*. Other differences related to organizational influences elicited through the domains of *beliefs about consequences* and, to a lesser extent, *social influences* and *social/professional role and identity*. Inquiries into the perceived influence of organizations were discussed in the context of quality-improvement strategies offered by the provider network, such as continuing education seminars, posting of the diagnostic-imaging guidelines on the organization’s website, and educational letters. Lastly, Canadian participants admitted ordering routine back x-rays for a majority of new patients. In contrast, American chiropractors generally reported referring fewer than 50% of acute low-back pain patients ( *nature of the behavior*).

**Table 2:** Distribution of utterances perceived to influence x-ray–ordering practice comparing American and Canadian participants

	**USA**				**Canada**			
**TDF domains**	**Reduce**^**a**^**(%)**	**Increase**^**b**^**(%)**	**Noinfluence (%)**	**Utterances (N)**	**Reduce**^**a**^**(%)**	**Increase**^**b**^**(%)**	**No influence (%)**	**Utterances (N)**
*Nature of the behaviors*	43 (61.4)	22 (31.4)	5 (7.1)	70	2 (11)	13 (72)	3 (17)	18
*Skills*	0 (0)	0 (0)	1 (100)	1	0 (0)	0 (0)	0 (0)	0
*Beliefs about capabilities*	62 (73.8)	21 (25)	1 (1.2)	84	8 (36)	10 (45)	4 (18)	22
*Motivation & goals*	25 (59.5)	12 (28.6)	5 (11.9)	42	3 (38)	5 (63)	0 (0)	8
*Beliefs about consequences*	73 (31.3)	136 (58.4)	24 (10.3)	233	75 (74)	20 (20)	6 (5.9)	101
*Environmental context & resources*	10 (40)	15 (60)	0 (0)	25	6 (38)	10 (63)	0 (0)	16
*Social influences*	29 (27.1)	60 (56.1)	18 (16.8)	107	18 (86)	2 (9.2)	1 (4.8)	28
*Emotion*	1 (25)	1 (25)	2 (50)	4	0 (0)	0 (0)	0 (0)	0
*Knowledge*	20 (40)	20 (40)	10 (20)	50	19 (53)	13 (36)	4 (11)	36
*Memory, attention, & decision process*	39 (92.9)	3 (7.1)	0 (0)	42	18 (72)	7 (28)	0 (0)	25
*Social/professional role & identity*	34 (33.3)	48 (47.1)	20 (19.6)	102	1 (3.2)	19 (61)	5 (16)	31
*Behavioral regulation*	61 (100)	0 (0)	0 (0)	61	30 (100)	0 (0)	0 (0)	30
**Total**	397 (48.4)	338 (41.2)	76 (9.3)	821 (72.1)	180 (58)	99 (33)	23 (7.5)	308 (27)

^a^Utterances perceived to reduce x-ray utilization (facilitators). ^b^Utterances perceived to increase x-ray utilization (barriers).

## Discussion

### Summary of findings

Focus groups using the TDF [58] identified the complex interplay between chiropractors’ beliefs about implementing diagnostic-imaging guidelines for adult spinal disorders in private practice. Our findings were consistent with previous research exploring factors influencing general practitioners’ [33,38,43,73-79] and chiropractors’ [5,19,47] use of guideline recommendations. Factors perceived to strongly influence lumbar x-ray–ordering practice clustered in five theoretical domains: *beliefs about consequences* (consequence of ordering x-rays and attitudes about the guidelines and professional experiences), *social/professional role and identity* (professional role, norms, boundary, autonomy, professional dignity or wanting to do the right thing, and agreement), *social influences* (influence from formal training, colleagues, publication), *beliefs about capabilities* (particularly when the patient’s diagnosis is unclear), and *knowledge* of the evidence base.

Having very few utterances coded in the *skills* domains in our study could be due to a number of reasons, including the low relevance of the domain as x-ray ordering differs from performing highly technical procedures, the nature of the questions themselves, coders’ interpretation of the domains and associated constructs, and coding within multiple domains. Only four utterances were elicited in the domain of *emotion*. Possible reasons for this low number of utterances include the following: (1) seeing back pain patients with a range of pain and disability levels may fail to elicit strong emotional reactions after several years in practice as one gains self-confidence ( *beliefs about capabilities*) and (2) focus groups may not be well suited to assess emotions among a group of healthcare professionals. Past reviews of barriers to guidelines and implementation have grouped attitudinal and emotional barriers together [33,78]. In the current study, statements implying lack of self-efficacy, confidence, sense of authority, and accurate self-assessment were classified under the TDF domain of *beliefs about capabilities*.

### Influence of professional identity

Heterogeneous and contradictory beliefs were expressed by participants about professional identity and cultural authority (*social/professional role and identity*). We observed disagreements among chiropractors about the scope of practice, professional autonomy, choice of lexicon (concept of chiropractic subluxation), and role of evidence. Professional identity remains an important source of tension between chiropractors [65,80,81], with paradigm differences (experiential vs evidence-based practice) likely driving other domains toward intention to manage back pain with or without x-rays. Such influence seems particularly important on the domains of *beliefs about consequences* (practice style, including x-ray–driven techniques), *social influences* (choice of literature and continuing education seminars), *knowledge* (guideline agreement), *memory attention and decision making* (taking x-rays if results are likely to change treatment protocols), and *nature of the behaviors* (ordering x-rays routinely or only in presence of red flags). A recent review of factors influencing health professionals’ intentions and behaviors found *social/professional role and identity* to be a substantial determinant of intention [82]. The domain of *social/professional role and identity* may act as a mediator of x-ray ordering among chiropractors. This will be further considered in a predictive study.

### Geographical variations

The main differences observed between US and Canadian chiropractors included perceived threat of litigation and HMO incentives to conform with evidence-based practice, two factors known to influence adoption of guidelines in general [47,73,75,83] and utilization of imaging studies in particular [84]. Perceptions of organizational influences, reimbursement system, incentives for particular procedures, resources available to help implement CPGs, and logistics were elicited through the domains *beliefs about consequences* and, to a lesser extent, *social influences* and *social/professional role and identity*. For instance, maintaining a higher tier was deemed important as it provided increased practice latitude by reducing the volume of paper work needed to justify ordering of spine x-rays. Providers’ perceptions of the ASH tier administrative system fell under the domain of *beliefs about consequences.* Corresponding constructs (reinforcement/punishment/consequences, incentives/rewards, and sanctions/rewards) may be important to consider when designing a behavior-change intervention among chiropractors enlisted with networks of providers and HMOs.

### Study limitations

While this study has provided valuable insight into the factors that may influence routine x-ray–ordering practices of chiropractors, there were several limitations. Recruitment was challenging, and two potential Canadian participants failed to show up. As a result, the number of participants in focus groups conducted was relatively small, and inclusion of other participants may have provided different beliefs either in favor of or against the targeted behavior [85]. This is particularly relevant to our secondary objective aiming to compare responses from American (n = 14) and Canadian (n = 7) chiropractors. However, the age, gender, and years in practice were representative of North American chiropractors. Further, the diversity of views, attitudes, and beliefs and the wide range of self-reported behavior (from rarely using x-rays to routinely doing so) reported by practitioners in four distinct geographical locations suggest that this may not be a major problem [86].

It is likely that other important barriers to guideline implementation would have surfaced had we also interviewed patients. Interviews of back pain sufferers suggested that lumbar x-rays were very important [87]. Patient’s views and expectations may considerably influence physician ordering and can be a barrier to appropriate use [38,41,43]. In the current study, chiropractors admitted ordering nonindicated x-rays to maintain trust, limit conflict, reduce patient anxiety, or protect professional dignity. Various strategies have been suggested to assist clinician and back pain patient encounter [87]. Engagement of patients in the decision process is another avenue to explore [88].

One important challenge when coding focus groups into the theoretical domains was the lack of clear definitions and the overlaps between multiple domains (*e.g.beliefs about consequences* and *motivations and goals*), rendering consensus difficult at times. In addition to using two independent coders with different professional backgrounds who reconciled differences at every step, findings were reviewed by a behavior psychologist (JJF) with in-depth knowledge of the TDF. Coding and subsequent agreement greatly improved after the first two transcripts. Validation of the TDF has resulted in a refined version addressing these shortcomings [89].

Although care was taken so participants would not get cues to answer in certain ways, agreement with other participants for fear of feeling marginalized by colleagues or to please the interviewer is a known weakness of focus groups. To account for social desirability, the interviewer asked participants to clarify nonverbal communication and coders considered patterns (*e.g.*, changed or reversed statements after hearing from others, recurrent comments, and themes supported or rejected by more than one participant) when linking utterances with specific beliefs. Discussions after each focus group suggested the interview guide did not feel repetitive and questions relating to domains felt relevant to the participants. Furthermore, the frequency of responses throughout focus groups (Table 1) indicated that participants remained engaged despite the number of questions asked (43 with prompts) and associated length of focus group interviews (around 90 minutes).

## Conclusion

Very few studies have attempted to examine potential barriers and facilitators to implementing guidelines among chiropractors using the TDF. Our study provides new insight into beliefs of chiropractors with respect to managing back pain without x-rays and theory-based factors likely to influence compliance. Adherence to diagnostic-imaging guideline recommendations appears to be influenced by a number of factors likely to either increase or decrease ordering of lumbar x-rays. Relevant TDF domains included *beliefs about consequences*, *social/professional role and identity*, *social influences*, *belief about capabilities*, and *knowledge*. These domains appeared to be important due to the high frequency of beliefs, presence of conflicting beliefs, and evidence of strong beliefs likely to impact the behavior. Study findings can be used to inform the development of a theory-based predictive survey to further explore determinants of routine lumbar x-rays for uncomplicated back pain and test whether *social/professional role and identity* is a mediator of x-ray ordering among chiropractors. Results may also assist in designing a tailored intervention to improve guideline adherence.

## Competing interests

AES is an associate editor of *Implementation Science*; JMG is a member of the Editorial Board of *Implementation Science*.

## Authors’ contributions

JMG, JJF, AES, and The Canada Prime Plus team conceived the original study and acquired funding. AEB adapted the study design, contributed to the development of the interview guide, conducted all interviews, and analyzed and interpreted the data. AMP contributed to the development of the interview guide, and analyzed and interpreted the data. JJF interpreted the analysis. JMG contributed to the development of the interview guide and supervised the research group. AEB wrote the manuscript, and all authors commented on the sequential drafts of the paper and agreed upon the final manuscript.

## Authors’ information

AEB has clinical training in nursing and chiropractic with over 15 years of clinical experience and holds a faculty position in a chiropractic department of a public university. He holds a CIHR Fellowship in the area of Primary Care and a KT-Canada Student Fellowship. AMP has graduate training in psychology and knowledge translation research and has significant experience in conducting and coding interviews with healthcare professionals using the TDF. JMG holds a Canada Research Chair in Health Knowledge Transfer and Uptake. AES held the Canada Research Chair in Interdisciplinary Healthcare Teams when this manuscript was originally being prepared. The Canada PRIme Plus team is an international collaboration of researchers consisting of health services researchers, health psychologists, and statisticians. The Canada PRIme Plus research team includes Jeremy Grimshaw, Melissa Brouwers, Jill Francis, Gaston Godin, Jan Hux, Marie Johnston, Louise Lemyre, Marie-Pascale Pomey, Anne Sales, and Merrick Zwarenstein. The views expressed are those of the authors.

## Support

This study was funded by CIHR.

## Supplementary Material

Additional file 1 Summary list of potential barriers and enablers identified in the literature. Click here for file

Additional file 2 Interview topic guide for focus groups. Click here for file

Additional file 3 Elicited beliefs within TDF domains. Click here for file
