# Supplementary material for: Identifying factors likely to influence compliance with diagnostic imaging guideline recommendations for spine disorders among chiropractors in North America: a focus group study using the Theoretical Domains Framework
Source: Implement Sci. 2012 Aug 31;7:82. doi: 10.1186/1748-5908-7-82 (PMC3444898; doi:10.1186/1748-5908-7-82)
Supplement: Additional file 1 — Summary list of potential barriers and enablers identified in the literature. [file 1748-5908-7-82-S1.docx]

**Additional File 1 – Summary list of potential barriers and enablers identified in the literature**

**General categories based on Cabana’s framework***

| **1. Professional factors** |
| --- |
| 1.1 Knowledge about the guidelines |
| - lack of awareness |
| - familiarity (volume of information, time needed to stay informed, critical reading skills) |
| 1.2 Attitudes |
| - lack of agreement with specific guidelines (interpretation of evidence, applicability to patients, lack of confidence in guidelines developer, perceived risk/benefits, costs) |
| - lack of agreement with general guidelines (not applicable to practice population, biased synthesis, not practical, too cookbook, decrease autonomy, decrease doctors self-esteem) |
| - outcome expectancy |
| - perceived risks of liability |
| - negative attitude toward Health Maintenance Organizations |
| - reduces autonomy |
| - professional dignity, wanting to do the right thing |
| - wish to prevent referrals to another care provider |
| - reasons evoked for taking spine radiographs |
| - to rule out serious diseases/pathology/in the presence of red flags |
| - to screen for contraindications prior to spinal manipulation |
| - to identify the causative lesion(s) (misalignment/subluxation/dysfunction) |
| - to determine the parameter for the adjustment (eg, line of drive) or to establish a treatment protocol (biomechanical and/or postural analysis) |
| - if patient has filed a workers' compensation/automobile insurance claim |
| - pressure from peers, patients and organizations |
| - clinical uncertainty regarding patient presentation |
| - professional autonomy (reaching clinical decisions without interference) |
| - clinical ordering criteria, ease of test ordering |
| - inertia of previous practice, past behaviour and prior use of guidelines |
| 1.3 *Characteristics* |
| - age |
| - years in practice |
| - chiropractic school attended |
| - postgraduate diploma, educator |
| - Practice setting |
| - practice type (solo vs group practice) |
| - practice location (metropolitan vs rural setting) |
| ***2. Patients* characteristics** |
| - patient direct request |
| - fear of serious disease |
| - understanding of what the problem is and possible causes |
| - beliefs about x-ray usefulness |
| - satisfaction with care |
| - guideline recommendations perceived to be offensive to patients |
| - historical and physical findings (acute vs chronic, pain level & distress, co-morbidity), |
| **3. Environmental characteristics (system/process and support/resource)** |
| 3.1 Health Maintenance Organization (HMO) |
| - work structure, material contexts, work-related activities, relationships, work culture, type of management and policies |
| - organizational constraints (time pressure, lack of resources, work pressure) |
| - financial issues: cost to patient, to practice or lack of insurance coverage, inappropriate overall physician compensation or reimbursement, incentives for particular procedures |
| - limited support/poor logistics for implementation, training opportunities and communication |
| 3.2 Diagnostic services |
| - Onsite radiography / self referral |
| - limited access to radiology services (distance to imaging facility) |
| 3.3 Guidelines themselves |
| - - ease of use |
| - - convenience |
| - - comprehensiveness , complexity |
| - - trialability (easy to tried out) |
| - - ease of implementing into practice, requires specific resources |

* Cabana M, Rand C, Powe N, Wu A, Wilson M, Abboud P et al.. Why don't physicians follow clinical practice guidelines? A framework for improvement. JAMA 1999, 282:1458 - 1465.
