# Supplementary material for: Identifying factors likely to influence compliance with diagnostic imaging guideline recommendations for spine disorders among chiropractors in North America: a focus group study using the Theoretical Domains Framework
Source: Implement Sci. 2012 Aug 31;7:82. doi: 10.1186/1748-5908-7-82 (PMC3444898; doi:10.1186/1748-5908-7-82)
Supplement: Additional file 2 — Interview topic guide for focus groups. [file 1748-5908-7-82-S2.docx]

**Additional File 2.** **Interview topic guide for focus groups**

**List of abbreviations**

ASH: American Specialty Health Network

DIGASD: Diagnostic Imaging Guidelines for Adult Spine Disorders

HMO: Health Manage Organization

Red Flags: Indicators of serious pathology

RCT: Randomized Control Trial

**Diagnostic Imaging Guidelines Implementation Study**

I’d like to start by asking each one of you to state your name slowly for the benefit of the person who will be transcribing the interview. It would be useful if you could remember to say your first name before commenting during the early part of our discussion for the same reason.

We understand management strategies may vary somewhat depending on whether patients are within HMO’s or not. Because we are interested in getting a wide range of information, we would appreciate if you could share your views for both ASH patients and non-HMO patients. This will make our findings more relevant to non HMO colleagues.

The behaviour of interest for today’s discussion is « managing new patients with non-specific back and pain without taking spine x-rays ». I’m going to keep emphasizing this throughout the interview.

*I would now like to ask you to tell me in general how you might manage a new patient with spine disorders in your office. By that I mean your usual routine once the patient is with you in the examining room, questions you ask and the exam procedures you do to determine if x-rays are needed.*

- *Prompts: What do you feel you do well? What do you feel you could do better?*

*Thank you.*

Now for the rest of the interview, I have some slightly more specific questions. Some may seem repetitive, but please bear with me as the questions are derived from multiple theories on human behaviour and we are trying to identify which theory best applies in this area. I may also ask for clarification during the interview using probes such as: *‘What do you mean’; ‘Would you explain that’; ‘What were you thinking at the time’; ‘Take me through the experience’; ‘What skills are required to do so?’; ‘How and why do you use it?’*

***** For the purpose of the interview, ‘non-specific’ refers to*** ***patients with uncomplicated mechanical back pain that varies with time and activity with no neurologic deficits, fractures or indicators of potentially serious pathologies (i.e. red flags)***

**Nature of the behaviors**

1. In your practice, how often do you come across new patients with non-specific back pain?
2. What percentage of those patients do you do spine x-ray on?
   - *prompts: do you systematically screen your patients for the presence of red flags?, do you assess the patient motivation of undergoing x-rays or no x-rays and if so, how?, What do you usually say to patients who ask to undergo x-rays but where you find it is not clinically warrant? Do you follow-up to monitor patients’ progress after treating them without prior x-rays? What is the outcome usually?*

**Skills**

1. How easy or difficult is it to manage a new patient with non specific back pain without taking spine x-rays? Why?

- *Prompts: patient’s compliance, lack of training, complexity of cessation guideline, lack of counselling skills*

1. What skills are required to) manage a new patient with non-specific back pain without ordering spine x-ray?
   - *Prompt: how easy or difficult those skills are.*
2. How much expertise or experience do you think one needs to have to manage non-specific back pain in a new patient without taking spine x-ray ?
   - *prompt: history taking, physician exam*

**Beliefs about capabilities**

1. How confident are you that you can manage non-specific back pain in a new patient without taking spine x-rays despite any difficulty?
2. What problems/difficulties do you think you might encounter in managing non-specific back pain without any x-rays?
3. What could help you overcome these problems/difficulties?

- *Prompts: additional training, communication techniques, continuing education, educational material, online information. Please elaborate on whether or not you think communication skills are important for managing patients without taking x-rays? Why is that?)*

**Motivation and goals**

1. How important do you feel it is (i.e. priority) to manage a new patient with non-specific back pain without taking spine x-rays? (in relation to other tasks like history taking and examination or others)
2. How does this differ between HMO and non-HMO patients?

- *Prompts: Are there any incentives that motivate you to manage patients with non-specific back pain without taking any x-rays? - recommendations from ASH, from Regulatory body, colleagues, the medical community or patients themselves*

1. Would there be anything else that you want to do or achieve that might interfere with your practice with regard to managing a new patient with non-specific back pain without taking spine x-rays?
2. Are there any incentives that motivate you to manage a patient with non-specific back pain without taking spine x-rays?

- *Prompt: goals within yourself? external?*

1. Do you set goals for yourself or your practice with regard to managing a new patient with non-specific back pain without taking spine x-rays?

- *Prompt: goals within yourself? external?*

**Beliefs about consequences**

1. What are the benefits of managing a new patient with non-specific back pain without taking spine x-rays?

- *Prompt: to self, or to patients i.e. reducing ionizing radiation exposure & costs?, profession, healthcare organization*

1. Do you feel there are potential harms or disadvantages in managing patients with non-specific back pain without any x-rays?

- *Prompts: 1) screening to prevent rare possible complications associated with spinal manipulative therapy, 2) accurate prognosis; 3) patient preference and satisfaction; medico legal* concerns or political influence & administrative factors

1. Do you feel that the benefits of managing a new patient with non-specific back injury without taking spine x-rays outweigh the costs? Why or why not?
2. Is there any incentive/disincentive that you can think of that influence whether or not you manage a new patient with non-specific back pain without taking spine x-rays?

**Environmental context and resources**

1. What aspects of your environment (physical vs. resource factors) influence whether or not you are able to order spine x-rays for patients with non-specific back pain?

- *Prompt: resources available to help you manage non-specific back pain patients without taking x-rays (information pamphlet or posters to inform patients about potential risks of ionizing radiation exposure)?*

1. Do you think having onsite radiology equipment influence the decision to x-ray new patients with non-specific back pain?

**Social influences**

1. How might views/opinions of others (colleagues, patients, professional groups) influences your decision to x-ray patients with non-specific back pain?

- *Prompt: anyone else; in what circumstances*

1. Do organizations such as HMO’s influence whether or not you order x-rays for new patient with non-specific back pain without taking spine x-rays?

**Emotion**

1. Do patient emotions/apparent distress ever affect whether or not you order spine x-rays for patients with non-specific back pain?
2. Does managing patient with non-specific back pain without taking spine x-rays evoke an emotional response in you?

- *Prompt: Do your own emotions ever affect your decision to manage patients with non-specific back pain without taking spine x-rays?*
- *Prompts: fear of missing a significant pathology*

**Knowledge**

We have talked about some of the evidence; I’d also like to find out about your knowledge and use of guidelines:

1. Do you use any guideline to help you make informed decision about when to x-rays for patients with back pain?

- *Prompts: How do you use it? Why do you use it? What do you think of it?*

1. How do you use the guidelines? (i.e. what do you actually, physically do? Do you ever read the guidelines to check if a behaviour you performed was guideline-compliant?)
2. What other evidence are you aware of?
3. How well do you think you understand the evidence surrounding optimal use of x-rays for non-specific pain?
4. What are your thoughts about the recommendations proposed by the DIGASD?

- *Prompts: Do you believe it to be evidence-based? how did you use it? When did you use it? What do you think of it?*

1. Do you agree with the guidelines? i.e. are the guidelines representatives of the evidence (quality, appropriateness)?
2. What kind of additional information would most likely influence your changing your clinical management?

- *Prompts: RCT’s, Systematic reviews, discussion with colleagues, conferences/seminars*

**Memory, attention and decision processes**

1. What thought process might guide your decision to manage a new patient with non-specific back pain without taking spine x-rays?

- *Prompt: What goes through your mind?*
- *Prompt: Is this something you would have to think a lot?*

1. Is managing patients with non-specific back pain without taking spine x-rays something you would usually do?
2. In what situation, if any, might it be difficult to think of an alternative to using x-rays?
3. What rules of thumb do you use to reach a decision, if any?

- *Prompts: red flags, decision rules, guidelines…)*

**Social/professional role and identity**

1. Do you think it is an appropriate part of your job to manage new patients with non-specific back pain without taking spine x-rays?

- *Prompts:* *What are they and why? Do your colleagues generally agree with you on this issue?*

1. Do you sometimes feel constrained by guidelines? What about protocols?
2. Is there anything else about your professional role that influences you managing patients without referring for spine x-ray? (i.e. Consensus in the chiropractic profession)
3. Do you tend to practice using an x-ray driven techniques to establish a treatment protocol?

i.e. Upper cervical or rehabilitation techniques (biomechanical analysis)

- *Prompts: What are they and why?*

**Behavioural regulation**

1. If you’re thinking about changing your own practice to manage new patients with non-specific back pain without taking spine x-rays, how would you do it?

- *prompt: self change, practice level change, training, education*

1. What might you do In order to reduce the likelihood of needing spine x-rays?
2. Are there procedures or ways of working that might encourage you to manage a new patient with non-specific back pain without taking spine x-ray?
3. If you decide to manage with non-specific back pain without taking spine x-rays, how confident are you that your associates can carry this out?
4. The evidence from research suggests that x-rays are not useful for non specific back pain pain. With that in mind, in terms of using less spine x-rays:
   - *What might need to be done differently?*
   - *What would you do differently?*
   - *Who needs to do what differently, when, where, how, how often and with whom?*

That all the questions I had for you today. Has anything else occurred to you about this topic that we haven’t asked about?

Overall, what were your thoughts about the interview?
