# Supplementary material for: Identifying factors likely to influence compliance with diagnostic imaging guideline recommendations for spine disorders among chiropractors in North America: a focus group study using the Theoretical Domains Framework
Source: Implement Sci. 2012 Aug 31;7:82. doi: 10.1186/1748-5908-7-82 (PMC3444898; doi:10.1186/1748-5908-7-82)
Supplement: Additional file 3 — Elicited beliefs within TDF domains. [file 1748-5908-7-82-S3.docx]

**Additional File 3. – Elicited beliefs within TDF domains**

| **TDF domains** | **Specific beliefs** |
| --- | --- |
| *Nature of the behaviours* | 1. I avoid ordering x-rays unless the history reveals specific indicators of a serious condition 2. I use x-rays as a diagnostic tool (to rule-out pathologies, fractures or dislocation) or when patients fail to improve 3. I rarely order x-rays 4. 5-10% of my new patients are x-rayed. I tend to be fairly conservative on x-ray 5. 40-50% of my new patients with back pain are x-rayed 6. 50-80% of my new patients with non-specific back and are x-rayed 7. I routinely x-ray my new patients with non-specific back and are x-rayed |
| *Skills* | None identified |
| *Beliefs about capabilities* | 1. I'm confident that I can manage non-specific back pain without x-rays 2. I'm comfortable/uncomfortable I can manage non-specific back pain without x-rays 3. For appropriate patient management, I prefer to x-ray when I'm uncertain of the diagnosis 4. As a result of my experience, managing new patients with non specific back pain without taking spine x-rays is easy/harder 5. Workers’ Compensation patients already have x-rays before seeing me |
| *Motivation and goals* | 1. Patient centered care is important to me 2. Finding out the reasons why a patient wants to have x-rays done when I think they don't need it helps me better communicate with them. 3. I only order x-rays when I feel they are clinically indicated 4. Reduce patient radiation exposure is important to me 5. I’m motivated to adapt my practice based on new evidence 6. Ordering x-rays is important/not important to me |
| *Beliefs about consequences* | 1. I take x-rays to avoid risks of adverse effects for my patient 2. I take x-rays so I'm not responsible for causing adverse treatment effects (practitioner consequence) 3. I order/I don't order x-rays because of the risk of litigation 4. I take follow-up x-rays to help monitor patient conditions / I don't think follow-up x-rays are useful 5. Patients may/may not benefit from seeing their own x-rays to improve treatment compliance 6. When I can, I avoid repeating x-rays because of ionizing radiation exposure 7. Benefits of managing non-specific back pain without x-rays include: minimizing ionizing radiation exposure, reducing costs, minimizing adverse events from further investigation, avoiding labelling of patients, maintaining the highest Tier 8. HMO will restrict your autonomy if you don't conform to their standards 9. X-rays are/are not helpful for making a diagnosis in uncomplicated back pain 10. I agree/disagree with ordering routine spine x-rays 11. X-rays are low-costs and low risk procedures 12. Disadvantages in managing patients with non-specific back pain without any x-rays include: risk of missing a pathology or anomalies such as spondylolisthesis 13. Guidelines parameters should be respected 14. Guidelines are designed to further restrict practice 15. Restrict x-ray use for cost reduction by HMO may impact on the quality of care |
| *Environmental context & resources* | 1. Onsite imaging does/does not increase x-ray utilization rate for regular chiropractic patients 2. Not having onsite imaging may/may not: influence patient decision to consult, decrease patient compliance and delays care 3. I do/don't have signs informing of the risk of radiation exposure |
| *Social influences* | 1. Patient expectation may/may not influence my decision to order x-rays 2. Colleagues, opinion leaders, mentors or instructors do/do not influence my practice 3. My training did/did not influenced my x-ray utilisation rate 4. Patient emotion can influence whether or not I order x-rays 5. HMO's/institutions guidelines, protocols or requirements do/do not influence my decision to order x-rays 6. The literature I read influence my imaging practice |
| *Emotion* | 1. X-ray guidelines are a source of anxiety for providers as we need to conform with the HMO 2. I get worried when patients don't improve as expected, so I order x-rays |
| *Knowledge* | 1. I am/am not aware of x-ray guidelines for chiropractors 2. There are conflicting guidelines 3. I agree/disagree with the guidelines 4. The guidelines are the minimum standard to follow 5. There is/is not enough evidence to support the guidelines 6. The evidence upon which the guidelines are based is not clear |
| *Memory, attention, decision processes* | 1. The decision to order or not order x-rays is based on the patient history and the patient age 2. The decision to order or not order x-rays is based on the patient history and examination 3. The decision to order x-rays is based on the patient history, examination and my experience 4. The decision to order x-rays is based on my experience alone 5. High pain intensity and inability to examine the patient are indicators of the need for x-rays 6. I order x-rays if patients are not improving after 6-8 visits or 3-4 weeks of care 7. I take x-rays if results are likely to changes my treatment protocol 8. Experience has reduced the effort required for me to make the decision |
| *Social/professional role & identity* | 1. X-ray driven chiropractic treatments are/are not part of my practice 2. The schools I attended did/did not train me to take routine x-rays 3. Staying current is important for the profession's credibility 4. Appropriate ordering of x-rays may/may not improve the credibility of the chiropractic profession 5. My practice is/is not dictated by ASH Guidelines 6. I have/I don't have the autonomy 7. Chiropractors pick and choose the guidelines to best suit their practice 8. The guidelines mostly apply to those chiropractors with x-ray ordering behaviour at the extreme ends 9. Lack of standardization, insufficient knowledge and skills in the profession are problematic 10. Abuse exists in any profession 11. Routine x-rays is/is not acceptable in our profession 12. The Guidelines do/do not support my way of practice |
| *Behavioural regulation* | 1. Audit and Feedback may help improve individual behaviour 2. Having access to a mentor or an experienced clinician is a way to encourage best practice 3. Guidelines implementation should include long term strategies for sustainability 4. Introducing the guidelines and discussing risk/benefits during CME may help reduce x-ray utilisation rate 5. Patient decision tools may help reduce x-ray utilisation 6. Guidelines should be targeted at those who either never use x-rays or routinely use x-rays 7. Guidelines should be based on high level evidence 8. Guidelines should be objective and not biased 9. Guidelines adherence may improve if they are simplified, have key messages, are freely accessible and are presented as valuable tools for short-term care 10. Regulatory body may effective to reduce overuse of x-rays 11. Practice changes would have to be instituted by the schools as they're the ones setting standards of care regarding x-ray ordering 12. Sharing liability would help me adhere to guidelines recommendations |
